# Effect of Electrode Insertion Angle on Cochlear Implantation Outcomes in Adult and Children Patients with Sensorineural Hearing Loss

**DOI:** 10.1155/2022/9914716

**Published:** 2022-08-23

**Authors:** Ting Fan, Meng-Ya Xiang, Yang Li, Jia-Min Gong, Tao Wu, Yue Wang, Jin Xu, Yun-Feng Wang, Jian Li

**Affiliations:** ^1^ENT Institute and Department of Otorhinolaryngology, Eye & ENT Hospital, Fudan University, Shanghai 200031, China; ^2^Clinical Laboratory Center, Children's Hospital of Fudan University, National Children's Medical Center, Shanghai 201102, China; ^3^Shanghai Lishengte Medical Technology Co., Ltd., Shanghai 201318, China

## Abstract

**Purpose:**

To determine the role played by electrode insertion angle in cochlear implantation (CI) outcomes in adult and children patients with sensorineural hearing loss (SNHL).

**Methods:**

Adults (*n* = 10) and children (*n* = 19) with SNHL undergoing CI in a tertiary specialized hospital were retrospectively enrolled. The measurements were evaluated before and after CI surgery using sound field audiometry and speech recognition tests. Questionnaires were used to assess subjective benefits. Electrode insertion angles were determined using postoperative X-rays.

**Results:**

Both adult and children patients showed significant improvements in hearing, speech performance, and audiology and speech-related quality of life after CI. The angular insertion depths of adult and children group were 323.70 ± 43.57° and 341.53 ± 57.07°, respectively, showing no significant difference. In the adult group, deeper insertion depths were found to be strongly linked to lower postoperative pure tone thresholds at 12 months and higher postoperative disyllabic Word Recognition and Sentence Recognition Scores at 6 months (all *P* < 0.05). In the children group, deeper insertion depth had a positive correlation with postoperative monosyllabic Word Recognition Scores 6 and 12 months after CI surgery (both *P* < 0.05). Multiple linear regression models were constructed to predict disyllabic Word Recognition Scores at 6 and 12 months postoperatively in the children group, in which insertion angle, duration of hearing loss, and preoperative questionnaire result were identified as dependent variables.

**Conclusions:**

Greater angular insertion depths resulted in improved hearing and speech performances after CI. The benefits of greater angular insertion depths can be found in both adult and children patients and last for at least 12 months. Clinicians are expected to determine the optimal implantation direction during CI and ensure the insertion depth to improve the speech rehabilitation of patients.

## 1. Introduction

For children and adult patients with severe or profound sensorineural hearing loss (SNHL), cochlear implantation (CI) has become the preferred treatment for their hearing rehabilitation in recent decades [1, 2]. Despite the well-documented effectiveness of CI in general populations, the outcomes of individual patients are still highly variable [3]. Many factors have been found to be associated with CI outcomes, including both demographic factors such as hearing loss duration and preoperative speech outcomes [4, 5] and surgical factors such as mediolateral placement of electrodes and the depth of electrode insertion [6, 7]. The scalar position of the electrode, the electrode-to-modiolus proximity, and the electrode insertion depth seem to be the major factors influencing electrode location [8]. It is important to understand the influences of these factors on CI outcomes, which is helpful for patient counseling and surgical practices in clinical practice.

Although many studies have demonstrated the significant impacts of electrode insertion depth/angle on patient outcomes postoperatively, the opinions differ. Generally, larger insertion angles are associated with more favorable speech performance, primarily due to improved electric stimulation of the apical region of the cochlear [9–12]. However, a study reported no correlation of insertion depth with speech perception in 100 adults with postlingual deafness [13]. Theoretically, inserting the CI electrode array deep into the cochlear apex to stimulate the complete spiral ganglion neurons covering the deeper region can enhance frequency alignment, allowing for a better experience of bass [14]. Some other studies have suggested that the greater the insertion angle, the more severe trauma during CI surgery, affecting postoperative word recognition of patients [15–17]. Based on the theory, deep insertion of the electrodes may cause confusion in the frequency and pitch of the ear tip [18], with a high risk of damage to the cochlear structure, possibly resulting in residual hearing loss and reduced stimulation of the basal rotation [17]. In previous studies, intergroup comparisons usually involved different device types [9, 11, 13, 17], and the variance in electrode size, shape, rate of stimulation, and signal coding strategy may bias research conclusions as probable confounding factors. Moreover, age at the time of implantation has been found to influence the relationship between insertion angle and CI outcomes. For example, Finley and Skinner found that after controlling for age, the inverse association between electrode depths and postoperative word recognition performance was no longer significant [17].

The present study is aimed at determining the role played by electrode insertion angle in CI outcomes. This study addressed the shortcomings of previous studies by using the same device from one single manufacturer and grouping the participants by age, thus evaluating potential differences between children and adults while keeping device variables constant.

## 2. Materials and Methods

### 2.1. Research Participant Selection

A retrospective case note review was performed to identify patients who underwent CI surgery at a tertiary specialized hospital from January 2016 to April 2022. Patients were included according to the following criteria: (1) diagnosis of bilateral severe or profound SNHL, (2) patients who underwent unilateral CI surgery with Shanghai Lishengte LCI20PI microcurved electrodes, and (3) those with smooth CI surgery according to the Chinese Medical Association guideline [19]. On the contrary, patients were excluded based on the following criteria: (1) patients with congenital or acquired inner ear pathologies, (2) patients who already used one hearing implant in one of their temporal bones, and (3) those whose postoperative cochlear images were obtained more than 3 days after surgery. Finally, 29 patients were enrolled. This study has obtained approval from the Institutional Review Board with an exemption of informed consent (Approval No. 2022050).

### 2.2. Demographics and Operative Characteristics

Patient demographics were retrospectively recorded, including age at implantation, sex, self-reported duration of hearing loss, cause of hearing loss, and preoperative use of hearing aids and duration of use.

The implantation of LCI20PI electrodes was performed using the traditional transmastoid approach, involving the procedure of antromastoidectomy and posterior tympanotomy through the facial recess. The round window niche was drill-grinded to expose round window membrane, followed by the insertion of the electrode into the scala tympani to stimulate spiral ganglion cells.

### 2.3. Electrode Insertion Angle

**Figure 1:**
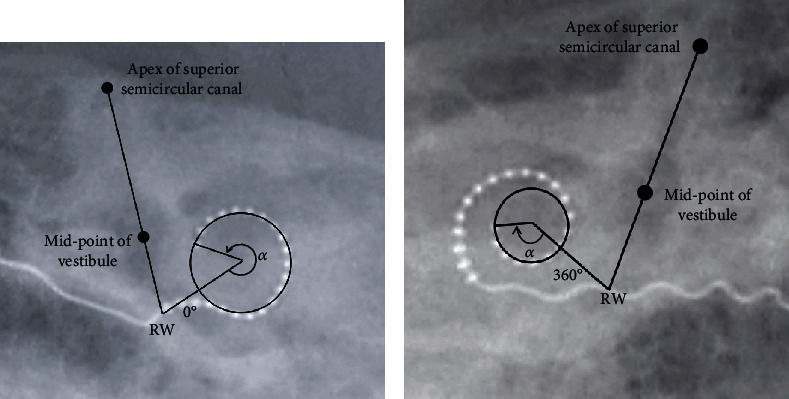
Measurement of electrode insertion angle. The line going through the apex of the superior semicircular canal and the vestibular aqueduct midpoint crosses the electrode array at the estimated round window (RW). (a) The line going through the RW to the center of first turn of the spiral is used as the 0-reference line. (b) In cases with deeper electrode insertion depth, the line going through the RW to the center of second turn is used as the 360-reference line.

The electrode insertion angle was determined by postoperative X-rays (modified Stenver's view). Two anatomic features, namely, the vestibular aqueduct midpoint and the apex of the superior semicircular canal, were identified. A line drawn through the two points served as a reference line for analysis, as it passed close to the round window (RW). The RW was determined by referring to the method described by Cohen et al. [20] and Xu et al. [21]. The electrode insertion angle belonging to the first turn of the spiral was computed from the line connecting the center of this first turn and RW and used as 0-reference line (Figure 1(a)). In other cases with deeper electrode insertions, the electrode insertion angle belonging to the second turn of the spiral was computed from the line connecting the center of this second turn and RW and used as the 360-reference line (Figure 1(b)).

### 2.4. Audiometric Performance

Audiometric assessments were performed in all patients, including pure tone audiometry evaluating sound field pure tone average (PTA) thresholds at 0.5, 1, 2, and 4 kHz, speech perception tests, and subjective questionnaires. All the assessments were tested before, as well as 6 and 12 months after CI surgery, except that the subjective questionnaires 6 months after CI were missing.

For the adult group, the speech recognition assessments included monosyllabic/disyllabic Word Recognition Score (mWRS/dWRS) and Sentence Recognition Score (SRS) in quiet using Mandarin Speech Test Materials (MSTMs) [22]. The speech materials were presented at 65 dB SPL. The subjective questionnaire used was the Categories of Auditory Performance (CAP) [23], an instrument evaluating hearing abilities from eight categories (0-7).

For the children group, the speech recognition assessments included mWRS and dWRS based on the Standards and Methods of Auditory and Language Skill Assessment of hearing-impaired children [24]. The speech materials were presented by the tester at the level of the usual voice. Subjects responded by choosing the picture on the picture set or by repeating the word they heard. The assessment of the development of auditory behavior in children was made using the Meaningful Auditory Integration Scale or the Infant-Toddler Meaningful Auditory Integration Scale (MAIS/IT-MAIS) [25].

### 2.5. Statistical Analysis

Measurement data and count data in this study were presented by mean ± SD (min, max) and *n* (%), respectively. A *t*-test or a Mann-Whitney test was adopted for between-group comparisons, depending on the features of the data set. The Pearson or Spearman analysis was performed to evaluate the correlations of the variables with cochlear implant outcomes. Multiple regression was carried out to determine the predictive factors of speech performance measures of interest: (1) angular insertion depth and (2) dWRS. All statistical analyses were conducted with IBM SPSS Statistics, v28 (IBM Corp., Armonk, NY), with *P* < 0.05 as the significance level.

## 3. Results

### 3.1. Demographic Characteristics

Nineteen pediatric patients, with an age range of 1-6 years (mean: 3.58 years), were included in the children group. Ten patients were included in the adult group aged 34.70 years on average, with an age range of 23-44 years. Demographic measures for both groups are presented in Table 1. A statistically longer self-reported duration of hearing loss was reported in the adult group. The majority of patients enrolled used hearing aids before implantation, with a statistically longer duration of use in the adult group, consistent with previous findings. Although the etiology of hearing loss in most patients was unknown, it seems to be mainly caused by acquired factors in adult patients and genetic factors in pediatric patients.

### 3.2. Cochlear Implant Performance

**Figure 2:**
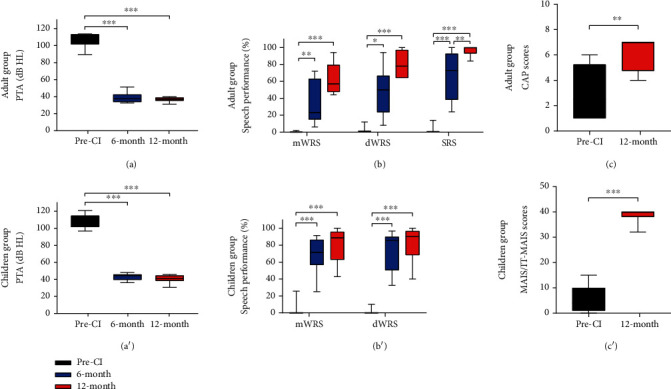
Cochlear implant performance in adult and children groups. (a) PTA in adult group at different time points. (b) Speech performance in adult group. (c) CAP scores in adult group. (a') PTA in children group at different time points. (b') Speech performance in children group. (c') CAP scores in children group. CI: cochlear implantation; PTA: pure tone average; mWRS: monosyllabic Word Recognition Score; dWRS: disyllabic Word Recognition Score; SRS: Sentence Recognition Score; CAP: Categories of Auditory Performance; MAIS/IT-MAIS: Meaningful Auditory Integration Scale or the Infant-Toddler Meaningful Auditory Integration Scale. ^∗^*P* < 0.05, ^∗∗^*P* < 0.01, and ^∗∗∗^*P* < 0.001.

The preoperative sound field PTA of adult and children groups was 109.69 and 109.38 dB HL, respectively, showing no significant difference. The PTA at 6 and 12 months after CI surgery reduced significantly compared to the pre-CI level in two groups (Figures 2(a) and 2(a')). The WRS scores were 0 in most patients in two groups, regardless of speech test materials. Significant improvement was observed in speech performance at 6 and 12 months postoperatively compared to the pre-CI level in two groups (Figures 2(b) and 2(b')). Notably, the SRS at 12 months was 25% higher than at 6 months in the adult group (*P* < 0.01), suggesting that SRS may better reflect the long-term effect of CI in adult patients. Subjectively, the CAP score improved significantly from a pre-CI level of 3.1 ± 0.64 to 7.0 ± 2.25 12 months after CI in the adult group, and the MAIS/IT-MAIS score improved significantly from a pre-CI level of 7.17 ± 1.53 to 40 ± 3.0 12 months after CI in the children group (Figures 2(c) and 2(c')). The above results showed that both adult and children patients obtained significant improvements in hearing, speech performance, and audiology and speech-related quality of life after CI.

**Figure 3:**
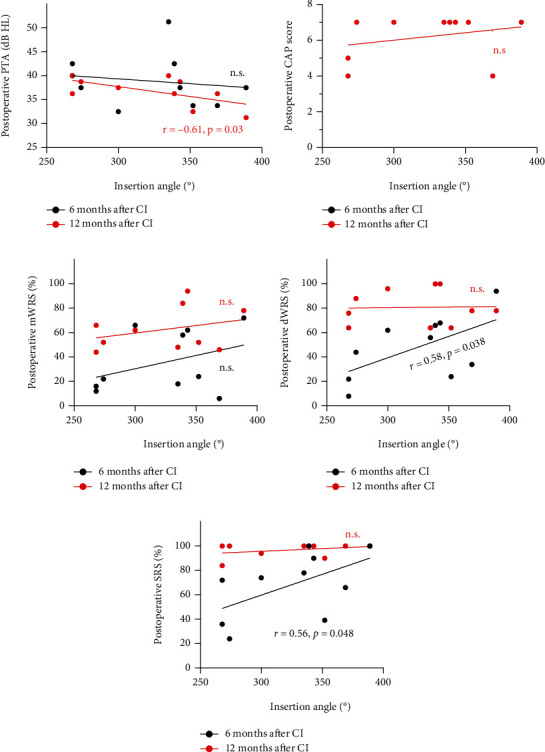
Correlation of insertion angle with cochlear implantation (CI) outcomes in the adult group. Correlations of insertion angle with postoperative PTA (a), CAP scores (b), mWRS (c), dWRS (d), and SRS (e). PTA: pure tone average; CAP: Categories of Auditory Performance; mWRS: monosyllabic Word Recognition Score; dWRS: disyllabic Word Recognition Score; SRS: Sentence Recognition Score; n.s: no significance.

**Figure 4:**
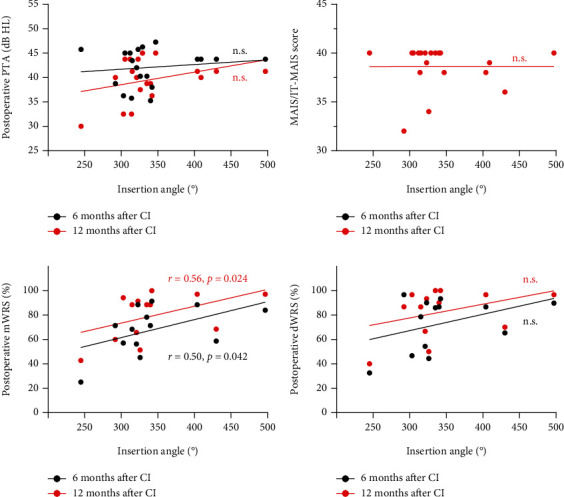
Correlation of insertion angle with cochlear implantation (CI) outcomes in the children group. Correlations of insertion angle with postoperative PTA (a), MAIS/IT-MAIS scores (b), mWRS (c), and dWRS (d). PTA: pure tone average; MAIS/IT-MAIS: Meaningful Auditory Integration Scale or the Infant-Toddler Meaningful Auditory Integration Scale; mWRS: monosyllabic Word Recognition Score; dWRS: disyllabic Word Recognition Score; n.s: no significance.

**Figure 5:**
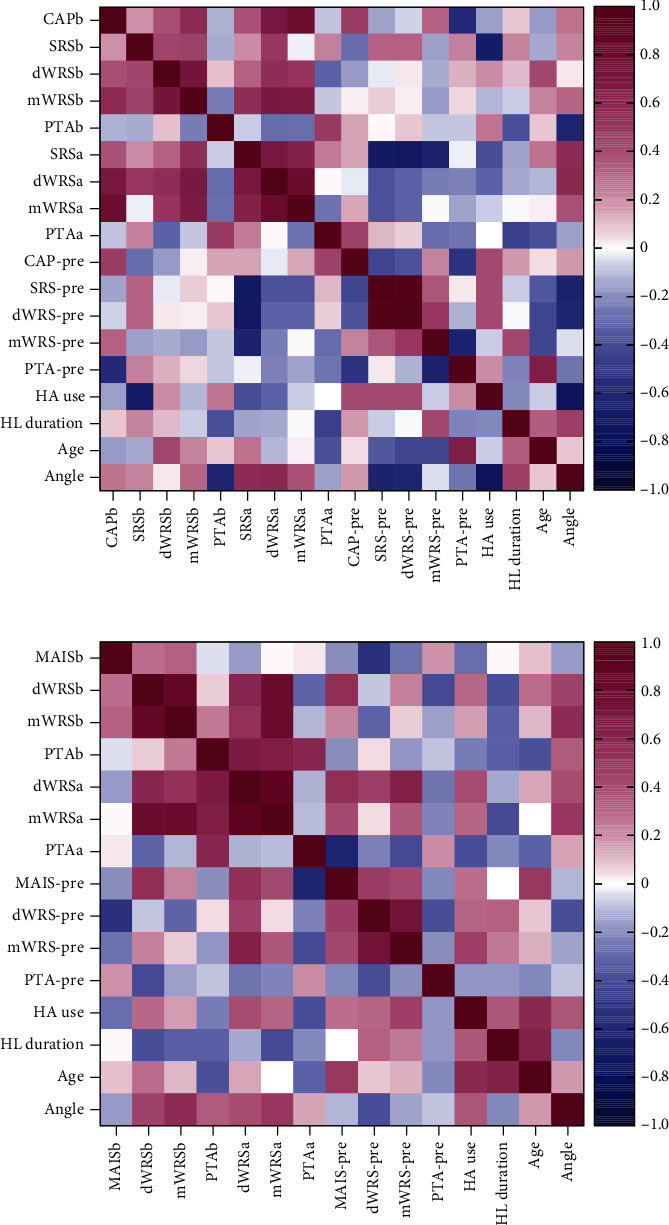
Correlation between independent variables and cochlear implantation outcomes in the adult group (a) and children group (b). PTA: pure tone average; mWRS: monosyllabic Word Recognition Score; dWRS: disyllabic Word Recognition Score; SRS: Sentence Recognition Score; CAP: Categories of Auditory Performance; MAIS/IT-MAIS: Meaningful Auditory Integration Scale or the Infant-Toddler Meaningful Auditory Integration Scale; HA: hearing aids. The suffix “-a” indicates data after 6 months of cochlear implantation and suffix “-b” indicates 12 months. Values represent the Pearson or Spearman correlation coefficients between features.

The angular insertion depth showed no statistical difference, with that in adult and children groups being 323.70 ± 43.57° and 341.53 ± 57.07°, respectively. In adult patients, deeper insertion depths were correlated with better postoperative hearing abilities in general (Figure 3). Specifically, the insertion depth showed significant negative correlation with postoperative PTA at 12 months and significant positive correlation with postoperative dWRS and SRS at 6 months (all *P* < 0.05). In the children group, deeper insertion depths had a positive correlation with postoperative speech performance, especially the mWRS at 6 and 12 months postoperatively (both *P* < 0.05) (Figure 4). Correlation analysis between other independent variables and CI outcomes was also performed and can be seen in Figure 5.

### 3.3. Prediction of Postoperative dWRS

The primary CI outcome was dWRS. Demographic variables that could predict postoperative dWRS were identified by the linear multiple regression analysis. It was found that the angle of insertion was correlated with the postoperative speech recognition in both groups, so the insertion angle was included as an independent variable in the regression analysis.

For the adult group, age at implantation and duration of hearing loss that were investigated as probable confounding factors and preoperative speech performance were used in the regression analysis (Table 2). Although the models of postoperative dWRS were not significant, the duration of hearing loss (*t* = −2.86, *P* = 0.035) and angle of insertion (*t* = 3.68, *P* = 0.014) may be significant factors to predict the postoperative 6-month dWRS. Besides, age at implantation had significant effects (*t* = 2.82, *P* = 0.037) on postoperative 12-month dWRS, indicating its potential to be a predictor for the long-term postoperative speech performance.

**Figure 6:**
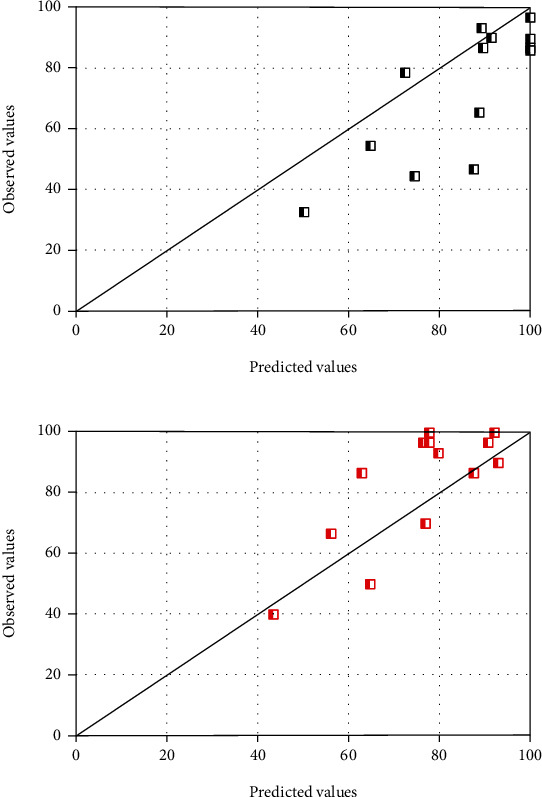
Individual prediction results of postoperative disyllabic Word Recognition Scores 6 months (a) and 12 months (b) after cochlear implantation in the children group.

For the children group, the other two independent variables (duration of hearing loss and preoperative MAIS/IT-MAIS score) were used in the analysis.  Table 3 illustrates the results by presenting analysis outcomes of two three-parameter models. The results showed that the angle of insertion and MAIS/IT-MAIS were helpful in obtaining regression equations that can predict postoperative speech perception. The predicted postoperative 6-month and 12-month dWRS were then plotted against their empirical values (Figure 6**)**. These results reflect the relatively even contributions made by the scores of preoperative MAIS/IT-MAIS and angle of insertion to the regression equation for postoperative speech perception in children group.

## 4. Discussion

The present research was designed to determine the correlation of angular insertion depths with cochlear implant performance. Postoperative plain film X-rays (modified Stenver's view) are necessary for patients undergoing CI to detect possible electrode kinking, confirm intracochlear position of active electrodes, and provide a reference in the event of postoperative slippage [26]. The function of cochlear implants is to directly stimulate neurons of the auditory nerve through electrical stimulation, bypassing damaged or missing structures. The modern CI nervous system selectively stimulates neuronal subpopulations using different electrodes inserted into a series of electrodes in the cochlear tympanic segment during surgery [27, 28]. It has been shown that it is more useful to specify electrode locations by angle than by length. Insertion angle, which represents insertion depth, facilitates the comparison of electrode array data under different intracochlear trajectories. For example, one array might follow the inner wall of the scala tympani, while another might follow the outer wall. Assuming the same overall angle of the cochlea, different insertion angles for the individual electrode array will result in different characteristic frequencies. In order to study patient speech performance and psychophysics, it is desirable and necessary to quantify individual electrode insertion angle [20].

We found that deeper insertion depths were associated with better CI outcomes. According to Hochmair et al. [29] and Hamzavi and Arnoldner [30], stimulating the cochlear apex can significantly enhance speech recognition, and distributing the contact points along the entire cochlea facilitates speech perception in a variety of environments. The benefits of deep electrodes are supported by morphological examination of the apical region. During electrical stimulation, spike initiation on peripheral processes adjacent to the basilar membrane contributes to enhanced channel selectivity. Yukawa et al. [31] also believe that greater insertion depths are linked to better speech perception due to a larger number of intracochlear sites available for electrical stimulation as well as a closer matching of the electrical with place-equivalent acoustic pitches.

Studies draw consistent conclusions in terms of increased insertion depths and improved speech perception performance [7, 31–34]. O'Connell et al. [33] studied 48 subjects implanted with lateral wall arrays and demonstrated a positive association between greater angular depths and better CNC performance (*r* = 0.48, *P* < 0.001) and the role of insertion angle as an independent predictor of increased CNC word scores after implantation. Strong evidence is supported by Buchman et al. [9] who prospectively and randomly assigned thirteen CI adults to receive either a standard (26.4 mm) or medium (20.9 mm) length electrode array; they found that in the early postoperative period (3 months and 6 months postoperatively), longer electrode insertions (and larger insertion angles) contributed to better speech perception performance. Another large-sample study was performed by Chakravorti et al. [7] who used the IRB-approved database of 220 CI ears to determine the correlation of audiological outcomes with factors like age, duration of CI use, device type, and electrode position; the angular depth of insertion was confirmed to be the most significant positional predictors of outcome for lateral wall arrays, while for the precurved arrays, full ST insertion and modiolar distance were the most significant factors. A systematic review of the association between the insertion angle and speech performance after 12 months postoperatively has also been performed, but no evidence-based conclusions have been drawn because of methodological flaws in all included studies. The present research found no significant correlation between angular insertion depths and postoperative 12-month speech performance, either. It is noted that the relationship between greater angular insertion depths and better speech performance was shown in the early postoperative period rather than in the long term. Likewise, De Seta et al. [35] demonstrated no association between insertion angle and speech performance after 1-year activation. Nor did Van der Marel et al. [13] find any relationship with postoperative speech outcomes at 1 and 2 years. This might be due to the plasticity of the brain, and learning and developmental effects should also be taken into account. Besides, speech discrimination is rising in the first year of postimplantation, when most CI implanters (approximately 90%) have attained stable speech recognition [3, 9, 36]. For the children group, children might need more time to adequate the CI, as most children undergoing CI have poorer speech perception than adults with this procedure. Theoretically, considering the positive results of CI children in speech and language development, this hypothesis indicates the improvement of social emotional ability after CI [37]. A systematic review of preschool and school-aged children undergoing CI found that the socioemotional development of CI children was neither average nor poor compared with their peers. Each child's task performance research may focus on different areas, including empathy and social interaction [38, 39].

According to the multiple linear analysis, the duration of hearing loss and insertion depth were associated with speech performance after activation, consistent with the finding of Yukawa et al. [31]. For the children group, preoperative IT-MAIS/MAIS scores and angle of insertion contributed to the regression equations predicting the speech perception postoperatively. Besides, a significant relationship between the dWRS at postoperative 6 months and the preoperative speech performance was found. Similarly, Van der Marel et al. [36] and Van Dijkhuizen et al. [40] reported that postoperative performance was associated with preoperative phoneme scores, which can support our findings.

Based on the above, surgeons need to construct the electrode approach in their mind, choose the exact cochleostomy site, and determine the best implantation direction, so as to ensure the expected insertion depth and improve patients' speech rehabilitation.

## 5. Conclusion

Greater angular insertion depths resulted in improved hearing and speech performances after CI. The benefits of greater angular depths can be found in both adult and children patients and last for at least 12 months. The insertion angle was an important factor in predicting postoperative dWRS among children patients. However, more data from a larger sample size need to be collected to confirm these findings.

## Figures and Tables

**Table 1:** Patient demographics.

Variable	Adult group (*n* = 10)	Children group (*n* = 19)	*P* value
Age at implantation (yrs)	34.70 ± 7.33 (23-44)	3.58 ± 1.77 (1-6)	__
Sex			0.089
Male	3 (30%)	*n* (63.2%)	
Female	7 (70%)	7 (36.8%)	
Duration of hearing loss (yrs)	7.00 ± 6.48 (1-18)	2.49 ± 1.75 (0.3-5.0)	0.060
Hearing aid use before implantation	10 (100%)	14 (73.7%)	0.075
Duration of hearing aid use (yrs)	6.07 ± 5.11 (1-18)	1.07 ± 1.27 (0-4)	__
Etiology^∗^			—
Sudden hearing loss	2 (20%)	0	
Hereditary hearing loss	0	2 (11%)	
Drug induced hearing loss	3 (30%)	0	
Unknown	5 (50%)	17 (89%)	

For numeric variables, measures are shown as mean ± SD followed by ranges in square brackets. ^∗^The etiology for both ears is the same for all the patients.

**Table 2:** Multiple linear regression analysis results on variables affecting postoperative disyllabic word recognition score in the adult group.

	6 months		12 months	
	*t*	*P*	*t*	*P*
Angle	3.680	0.014	1.980	0.105
Duration	-2.860	0.035	-1.620	0.166
Age	1.530	0.186	2.820	0.037
Speech performance-pre	2.080	0.092	2.430	0.059
Model significance (*F*, *R*^2^, *P*)	4.01, 0.57, 0.08		2.08, 0.32, 0.22	

**Table 3:** Multiple linear regression analysis results on variables affecting postoperative disyllabic word recognition score in the children group.

	6 months		12 months	
	*t*	*P*	*t*	*P*
Angle	2.630	0.017	2.580	0.030
Duration	0.090	0.929	-0.420	0.682
MAIS/IT-MAIS-pre	2.920	0.010	2.940	0.017
Model significance (*F*, *R*^2^, *P*)	4.45, 0.46, 0.035		4.90, 0.49, 0.027	

## Data Availability

The labeled datasets used to support the findings of this study are available from the corresponding author upon request.
